# The picture of undergraduate dental basic research education: a scoping review

**DOI:** 10.1186/s12909-022-03628-9

**Published:** 2022-07-23

**Authors:** Haiwen Liu, Zhuohong Gong, Chen Ye, Xuejing Gan, Shijie Chen, Lin Li, Yun Hong, Junqing Xu, Zhengmei Lin, Zetao Chen

**Affiliations:** Hospital of Stomatology, Guanghua School of Stomatology, Guangdong Provincial Key Laboratory of Stomatology, Sun Yat-sen University, 56 Lingyuan Road West, Guangzhou, 510055 Guangdong China

**Keywords:** Basic sciences, Curriculum infrastructure, Dentistry, Medical education research, Undergraduate

## Abstract

**Background:**

Undergraduate dental basic research education (UDBRE) is broadly regarded as an important approach for cultivating scientific research talent. This scoping review aims to summarize the current status of UDBRE in terms of educational goals, teaching program and content, assessment system, training outcomes, barriers, and reflections.

**Methods:**

The authors performed a systematic literature search in PubMed, Web of Science, and Education Resources Information Center (ERIC) to identify peer-reviewed articles written in English from their inception to January 29, 2021. Articles were reviewed and screened according to the inclusion and exclusion criteria. Related data from the included publications were then collected and summarized.

**Results:**

The authors searched 646 publications and selected 16 articles to include in the study. The education goals included cultivating five major dental basic research capabilities (*n*=10, 62.5%) and developing interest in basic research (*n*=2, 12.5%). As for the teaching program, the mentor-guided student research project was the most popular (*n*=11, 68.8%), followed by didactic courses (*n*=5, 31.3%), experimental skills training (*n*=1, 6.3%), and the combination of the above forms (*n*=3, 18.8%). However, the assessment system and training outcome diverged. Existing evidence showed that UDBRE reached satisfying education outcomes. Barriers included excessive curriculum burden (*n*=2, 12.5%), tutor shortage (*n*=3, 18.8%), lack of financial support (*n*=5, 31.3%), and inadequate research skills and knowledge (*n*=5, 31.3%).

**Conclusions:**

Although efforts were made, the variation between studies revealed the immature status of UDBRE. A practical UDBRE education system paradigm was put forward. Meanwhile, more research is required to optimize a robust UDBRE system with clear education goals, well-designed teaching forms, and convincing assessment systems.

## Background

According to the director of the US Office of Scientific Development and Research, “basic research” means advancing scientific knowledge and understanding of a topic or certain natural phenomenon, primarily in natural science [1]. Basic research is theoretical and focuses on general principles and testing theories and the importance of basic research in dentistry development is beyond question. Breakthroughs in dental basic research have profoundly advanced the diagnosis and treatment of dentistry by generating new ideas, principles, and theories and advancing fundamental knowledge of dentistry [1, 2]. For example, the formulation of the three primary factors theory (bacteria-diet-host) established the theoretical basis of prevention strategies such as plaque control and pit and fissure sealing [3]. The establishment of mechanobiology-based bone remodelling theories underpins the biologic basis of contemporary orthodontic therapy [4]. In short, dental basic research contributes substantially to the advancement of dentistry.

However, the current dental scientist talent pool is facing a shortage, and the competitiveness of dental talent is decreasing [5, 6]. The 2020 American Dental Education Association (ADEA) survey of dental school seniors showed that 85% of dental graduates chose private practice, rather than pursuing research careers [7]. Moreover, from 1999 to 2012, trends in the numbers of grant applications and awards to dentist-scientists point to an overall decline. The average age of first-time funded dentists was 52.7 years for females and 54.6 years for males [8]. Most dental practitioners are equipped with clinical skills, yet have relatively poor research abilities.

This trend leads to the reflection on the effectiveness of current dental education in cultivating dental research talent. As early as 1926, the Gies Report recommended that dental education should encourage and provide dental students with research opportunities within the optional dental curriculum [9]. “Undergraduate Dental Basic Research Education (UDBRE)”, concerning the topic of “basic research”, serves as an integral part and complement of undergraduate dental education [10, 11]. It includes not only laboratory-related training (RCR, western blot, etc.) but also the primary introduction of commonalities of research, including knowing what is a problem, how to raise a scientific problem, etc. [12]. UDBRE enhances the access, acceptance, and applicability of basic science for dental undergraduates [13] in various forms, including but not limited to didactic lectures [13–17], laboratory-based experimental courses [16], student research programs [13, 15, 18–23], etc.

UDBRE is broadly regarded as an important approach in training innovative dental researchers [23]. For individuals, UDBRE equips undergraduates with overall “basic research” capacities [10, 14, 15, 17, 22, 23], serving as fundamental tools to solve basic science problems and further achieve academic breakthroughs. In addition, UDBRE stimulates active learning and critical thinking [10] and sparks scientific interest [15, 23], leading dental students to reflect and discover basic science problem in daily clinical practice, and therefore, contribute to dentistry advancement. Over time, trained students, equipped with both clinical skills and adept “basic research” capacities, boost the scientist-dentist talent reserves and show a higher willingness to stay in school to continue an academic career as well as to teaching, which leads to the expansion of college staff and therefore relieves the current status of brain drain [13]. Supported by the government in policy and finance [24], UDBRE has become a new hot spot in dental education.

Dental clinical education has formed a mature training system, starting with didactic courses, then probation, internship, general training, and finally professional training to achieve educational goals at different stages [25, 26]. In contrast, UDBRE is still at a primary and immature stage. Most dental schools have not started student research programs or provide inadequate research programs due to various limitations [15]. The existing UDBRE education goals are vague, which may misdirect the proper setting of specific curricula. Thus, the current curriculum formats are diverse, and an optimized UDBREE system according to students’ step-by-step learning process has not yet been formed [10, 11, 13–23, 27]. Furthermore, the assessment methods vary. It is not yet clear which indicators can truly reflect the genuine effects of UDBRE, and the lack of an established optimized assessment system has also caused difficulties in curriculum design [13, 18, 19, 23]. It is unclear how the UDBRE is performed in different regions, including the content, teaching format and assessment methods. The training outcomes and the challenges in the implementation are also confusing. In addition, the immature development stage of UDBRE and the small number of related studies call for larger scale collection of information.

For these reasons, a scoping review was performed to systematically collecting information in the area, and identifying any existing gaps in knowledge to conclude the current picture of the UDBRE programs in terms of goals, content and teaching format, assessment, outcomes, barriers, and challenges. It is essential to establish an advanced education model of UDBRE and analyse it from a scientific perspective.

## Materials and methods

This scoping review was performed in accordance with the PRISMA Guidelines [28]. Three trained researchers conducted a systematic search in PubMed, Web of Science, and Education Resources Information Center (ERIC) databases. “Dental education”, “Undergraduate”, “Basic research”, and their synonyms were used as keywords (Table 1).

**Table 1:** The search strategy and keywords used with each database

Database	Search strategy and keywords	Number
PubMed	(((((dental OR dentist? OR stomatal?)) AND ((student? OR educate? OR school?))) AND ((basic sciences OR scientific research OR academic career?))) AND ((course OR mentorship OR curricula? or educate? OR program? OR educational methodology OR teaching methods))) AND ((undergraduate) OR (pre-postgraduate))	372
Web of Science	TS = ((undergraduate) AND (dental OR dentist? OR stomatal?) AND (student? OR educate? OR school?) AND (basic science OR scientific research OR research? OR academic career?) AND (course OR mentorship OR curricul? OR educate? OR program? OR educational methodology OR teaching methods)) OR TS = ((dental OR dentist? OR stomatal?) AND (basic science OR scientific research OR research? OR academic career?) AND (undergraduate) AND (course OR mentorship OR curricul? OR educate? OR program? OR educational methodology OR teaching methods)) OR TS = ((undergraduate) AND (dental OR dentist? OR stomatal?) AND (student? OR educate? OR school?) AND (basic science OR scientific research OR research? OR academic career?))	178
ERIC	(((((dental OR dentist? OR stomatal?)) AND ((student? OR educate? OR school?))) AND ((basic sciences OR scientific research OR academic career?))) AND ((course OR mentorship OR curricula? or educate? OR program? OR educational methodology OR teaching methods))) AND ((undergraduate) OR (pre-postgraduate))	96

### Eligibility criteria

The inclusion criteria were as follows: (1) all studies related to “Dental education”, “Undergraduate” and “Basic research” no matter curriculum forms, (2) English-language articles, and (3) articles published from their inception to January 29, 2021.

The exclusion criteria were as follows: (1) studies not focusing on one of “dental education”, “undergraduate” or “basic research”, (2) studies related to “dental hygiene” or “dental technology” were also excluded because these subjects were different from “dentistry” in curriculum, and (3) non-English written articles.

### Selection of sources of evidence

**Fig. 1:**
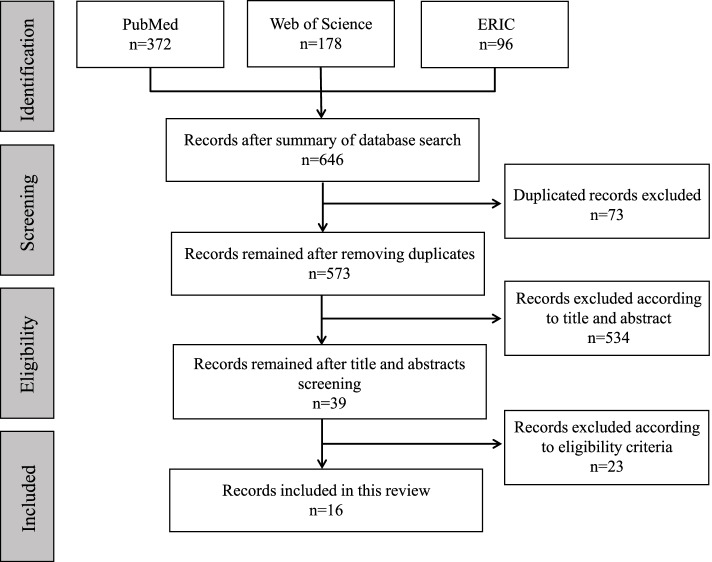
PRISMA flow diagram. PRISMA (Preferred Reporting Items for Systematic Reviews and Meta-Analyses) flow diagram shows the detailed process of information retrieval and literature screening

Three reviewers searched PubMed, Web of Science, and Education Resources Information Center (ERIC) databases, removed duplicate documents, and screened the articles independently according to the titles and abstracts. Then, each of the three reviewers screened the full texts of two-thirds of the retained articles, which means that each article was reviewed twice. Throughout the whole process, reviewers held meetings to address discrepancies and reach an agreement on the final included articles. The process of screening literature is summarized in a flow diagram (Fig. 1).

### Data charting process and synthesis of results

The authors extracted information from the included articles, includes basic information, education goals, teaching programs, assessment methods and indicators, educational outcomes, barriers, and main conclusions.

## Results and discussion

In total, 646 articles were obtained initially and 16 articles were included according to the inclusion and exclusion criteria (Fig. 1). The included articles have been gradually published since 2008. The duration of the education project in each article varied, and lasted for a maximum of 25 years. The basic information of the included articles is listed in Table 2.

**Table 2:** Summary of basic information, characteristics and main conclusions of included literatures

Author, year	Type of articles	Type	Location reported	Education goals	Curriculum forms	Teaching program	Assessment method	Assessment indicators	Educational Outcome	Barriers	Main conclusions
Divaris, 2008 [10]	Working group report	EGC, DP, IB	International	Overall competences	ECA	MgSRP	/	/	/	ECB, LFS	The group summarized students’ views on the academic environment and proposed the integration of research components into the curriculum by research projects.
Rushton, 2008 [6]	Review	IB	Britain	/	/	/	/	/	/	TS	The review pointed to problems affecting academic dentistry in the UK and the lack of academic staff.
Scott and de Vries, 2008 [13]	Quantitative research	DP, AS, SO	Canada	/	ECA	MgSRP	Oral presentation, thesis	The number of participants, career choice, GPA	Participants had better academic performance, won more prizes, and showed more willingness to further study and retention.	/	In the BSc Dent program, dental undergraduates did research in laboratories and completed with presentations and articles. The program is flexible in time without specific courses.
Scott, 2008 [27]	Report	EGC, DP, AS, SO	Canada	SRW, PSI	ECA	MgSRP	Competitions	Research competition awards	Some students won prizes in scientific research competitions for outstanding work.	/	This article shows the cases and experiences of distinguished members of the BSc Dent program.
Grossman, 2009 [18]	Quantitative research	DP, AS, SO, IB	South Africa	/	CC, EC	TC, MgSRP	Questionnaire, competitions, presentations, thesis	Self-assessment	Most agreed they would do research in the future and 1/3 students were satisfied with the research experience.	TS, LFS	South Africa combined research components into the undergraduate dental curriculum, including lectures, experiments, written assignments, and presentations. Students assist in established projects or undertake new studies individually.
Guven, 2011 [11]	Quantitative research	EGC, DP, AS, SO	Turkey	RD, SRW	ECA	MgSRP	Questionnaire	Self-assessment, number of funds, future career choices, GPA	Club members showed the improved ability of research design, experiment operation, and scientific report writing, with higher GPA, higher retention rates, and increasing funded projects.	/	409 different students presented 193 research projects in the Student Research Club. Students applied for research projects voluntarily and present their research in 10 to 15 minutes of speeches. Financial support was provided by each department. The club is proved to be beneficial to the academic career.
Franzén, 2013 [19]	Qualitative research	EGC, DP, AS	Sweden	BRPD, LR, SRW	CC	MgSRP	Thesis	/	/	/	Undergraduate research project in Swedish dental schools includes accomplishing experiment and writing papers under the teachers’ guidance. This project was integrated into the Swedish dental curriculum system.
Jeelani, 2014 [14]	Quantitative research	DP, AS, SO, IB	Pakistan	/	ECA	TC	Questionnaire	Self-assessment, attendance rate	59.2% of students knew how to plan a study, 46.7% knew how to write the article, 68.7% had research experience.	ECB, LFS	The dental school introduced a research methods course for third-year dental undergraduates. Most surveyed students complained about the lack of funds and academic load.
Franzén, 2014 [20]	Qualitative research	EGC, DP, AS, SO	Sweden	SRW	CC,	MgSRP	Thesis	/	Students focused on different topics but showed inadequate reflection on the link between research and clinic.	/	This study investigated the choice of topic and research method, and students’ awareness of the clinical significance of research results.
Ping, 2015 [15]	Quantitative research	DP, AS, SO, IB	China	/	ECA	TC, MgSRP	Questionnaire	Self-assessment	50% of non-participated students admitted they had no interest in scientific research, especially senior students.	SK, IG	Chinese dental undergraduates applied for student research programs or participated in research programs of teachers.
Habib, 2018 [29]	Quantitative research	AS, SO, IB	Saudi Arabia	/	/	/	Questionnaire	Self-assessment	Students showed mediocre attitudes toward scientific research.	ECB, TS, ISK	The survey found students faced barriers like curriculum burden, lack of interest and scientific knowledge, and faculty staff shortage.
Costa-Silva, 2018 [16]	Qualitative research	EGC, DP, AS, SO	Brazil	ET	CC	TC, EST	Experiment report, exam, oral presentations, questionnaire	Experiment lesson scores, experimental reports, self-assessment	Attended students had higher experimental course scores, and more content of methodology and literature support was found in their experimental report.	/	The Cell Biology course includes theoretical and experimental parts. Students focused on biocompatibility assay of dental biomaterials, searched the literature, conducted experiments, collected data, and finished experimental reports.
Kyaw, 2018 [21]	Qualitative research	EGC, DP, AS, SO, IB	Malaysia	BRPD, LR, RD, SRW	CC	MgSRP	Questionnaire	Self-assessment	Half of the students had moderate knowledge and attitude toward research and believed they can critically appraise literature to a certain degree.	LFS, ISK	The student research project was an integrated final-year curriculum in the college, and it was encouraged to publish their work. Barriers like lack of skills and knowledge, funds, and time were found.
Nieminen, 2020 [17]	Qualitative research	EGC, DP, AS, SO, IB	Malaysia and Finland	LR	CC	TC	Questionnaire	Selfassessment	80% of students perceived their literature retrieval skills were good or passable in Finland and Malaysia	ISK	The Malaysian schools conducted 12-week lessons on literature retrieval. The Finnish schools conducted courses on scientific thinking and the principles of scientific research.
Otuyemi, 2020 [22]	Qualitative research	EGC, DP, AS, SO	Nigeria	SRW	CC	MgSRP	Questionnaire, thesis	Self-assessment	The students understood the research project well and showed moderate satisfaction.	/	The last-year undergraduates should finish the scientific research project and thesis.
Yu, 2020 [23]	Qualitative research	EGC, DP, AS, SO, IB	China	BRPD, RD, ET, SRW, PSI	ECA	MgSRP	Questionnaires, oral presentations, midterm assessment	Funded projects, publications, participants, attendance rate, Self-assessment GPA,	Attended students improved ability in research design, experiment operation, and report writing, with increasing funded projects and published papers. They had higher GPAs and won more scholarships with a high satisfaction rate.	LFS, IG, ISK	In the two-year period undergraduate research program, the Office of Dental Education recommended advisors to students and provided funds and laboratory support. Guided by mentors, students accomplished the program and finished the midterm assessment and final report.

Abbreviations for reported type: *EGC* Education goals consensus, *DP* Description program, *AS* Assessment system, *SO* Study outcomes, *IB* Implementation barriers

Abbreviations for education goals: *BRPD* Basic research question discovery ability, *LR* Literature retrieval ability, *RD* Research design capability, *ET* Experimental techniques, *SRW* Scientific report writing ability, *PSI* Promoting scientific interest

Abbreviations for curriculum forms: *CC* Compulsory curriculum, *EC* Elective curriculum, *ECA* Extra-curriculum activity

Abbreviations for teaching program: *TC* Theoretical courses, *EST* Experimental skills training, *MgSRP* Mentor-guided student research projects

Abbreviations for barriers: *ECB* Excessive curriculum burden, *TS* Tutors’ shortage, *IG* Insufficient guidance, *LFS* Lack of financial support, *ISK* Inadequate basic research skills, and background knowledge

### Goals

Specific targets of UDBRE have been put forward around the ultimate goal: “cultivating dental research talent with basic research capabilities and strong scientific interest” [10, 11, 16, 17, 19–23, 27] (Table 2). According to the process of scientific research, the proposed target competencies are subdivided into five aspects: (1) Basic research question discovery ability. Students should put forward innovative scientific questions from the difficulties of dental clinical practice [19, 21, 23]. Creativity is also an important dimension [19]. (2) Literature retrieval ability. Undergraduates shall be capable of conducting literature retrieval, reviewing the progress of the research question, critical thinking on previous research, and proposing a hypothesis [17, 19, 21]. (3) Research design capability. This goal expects students to retrieve literature, think critically, apply theoretical knowledge [11], formulate clear aims [19], design protocols [23], integrate creative ideas, consider ethical principles [19], and conduct preliminary experiments [23]. Many student research programs also aim to cultivate the ability to obtain financial support (research funds, scholarships, etc.) by writing applications or oral presentations on their research projects [11, 23]. (4) Experimental techniques. The student should master basic laboratory techniques, obtain valid data and analyse experimental data [16, 23]. (5) Scientific report writing ability. It comprises data analysis [23], graph plotting, critical thinking, and scientific report writing (thesis, article, etc.) [11, 19–22, 27]. Academic communication is a further goal. After the complete training of UDBRE, students are expected to deepen their understanding of scientific knowledge and develop professional theory and practice [19], as well as to establish their competencies in problem-solving and teamwork [10]. In addition to cultivating capabilities, promoting scientific interest is also an important goal of UDBRE, which is ignored by many guidelines [23, 27].

Specific goals of UDBRE programs are proposed but divergence exists within studies. The cultivation of the above five major abilities and the promotion of scientific interest serve as ideal objectives of UDBRE. Clarifying the education target helps dental schools design specific education methods to fulfil the goals.

### Content and teaching format

The reported content and teaching format of UDBRE are diverse but have something in common. The authors identified four major forms (Table 2): (1) theoretical courses or lectures [14–18]; (2) experimental skills training [16, 23]; (3) mentor-guided student research projects [10, 11, 13, 15, 18–23, 27]; and (4) combination of above forms [15, 16, 18].

#### Dental basic research theoretical courses

A Brazilian dentistry school reported adding theoretical lessons before the experimental course. Compared with those who attended a single laboratory class, participants had more discussion and showed a deeper understanding of important science topics in the final reports [16]. Similar lessons have been reported [14, 15, 17, 18]. The dental basic research theoretical courses before the experimental lessons are helpful to contextualize basic research in dental courses, learn scientific knowledge and establish scientific thinking.

The teaching content of each study had different focuses, covering scientific research methods [14, 18], literature retrieval [17], laboratory safety [16], training of basic research thinking [14], data analysis, and paper writing [17]. Regrettably, no document recorded the textbooks or reference materials used. The specific teaching methods also had their own merits. Some were traditional didactic curricula [18], and others adopted novel methods, such as project-based learning [16] and problem-based learning [10].

Although it is important in systematic research knowledge enlightenment, theoretical courses in UDBRE have been reported in relatively few studies [14–18] (Table 2, *n* = 5). The teaching content and method of didactic courses were unclear and more efforts are needed to improve feasibility. The basic research process, academic norms, and primary skills of dental basic research should also be included in the teaching content.

#### Experimental skills training in UDBRE

Experimental skills training (Table 2, *n* = 6) is usually integrated into UDBRE, together with other programs [11, 13, 16, 18, 20, 23], rather than an isolated educational program. The most common situation is that undergraduates learn the experiment involved in their projects under mentorship [23]. Another situation is to combine experimental and theoretical courses [16]. Although the current method is feasible, students may lack systematic training, and acquire experimental skills occasionally and irregularly. Such scattered and nonstandard learning should be transformed into a systematic and well-designed course.

#### Mentor-guided student research project

The mentor-guided student research project is the most common among the included articles [10, 11, 13, 15, 18–23, 27] (Table 2, *n* = 11), in which dental students experience the research process under mentorship. It usually begins with dental scientific questions put forward by undergraduates, and then they design their own scientific research projects after preparation (literature research, protocol design, etc.). Subsequently, they apply for research funds, scholarships, or student research projects. If accepted, students need to complete experiments in the laboratory, analyse data, express critical reflections, write an article or a report and finally complete the student research project under the guidance of the tutors [11, 13, 15, 18, 19, 22, 23].

Most research topics focus on dentistry. For topics in the field of medicine, human sciences, or other professionals, students needed to discuss the contribution of their research findings to dental practice [19, 23], which may promote interdisciplinary research. In terms of specific disciplines, in clinical departments, orthodontics, oral surgery, periodontology, and restorative dentistry have been the most popular fields. While the most attractive basic science departments have been microbiology, biochemistry, and pathology [11].

Although interdisciplinary mentorship is practicable, the research topic should be within the field of dentistry due to its unique characteristics. However, many schools failed to popularize student research projects due to insufficient dental supervisors. In this situation, interdisciplinary research is acceptable because scientific research has commonalities.

#### Summary

As the results disclose, UDBRE has not yet been popularized worldwide but dental schools have become aware of the significance of UDBRE. Some schools have begun to make some efforts and have achieved preliminary results. Various forms of UDBRE have been established, such as theoretical courses, experimental skills training, and mentor-guided student research projects.

Compared with the mature clinical training model, dental basic research education has not been organized. There have not been many integrated projects of the above forms [15, 16, 18]. Most of them are short-term projects of up to two years [18, 23]. Long-term training projects have not yet appeared. Given all this, the next goal of UDBRE is to form a scientific and gradual education system.

There are differences between education forms. Which are better methods also remains unknown. How to organically integrate different education forms to maximize the effectiveness of education requires further study. Moreover, the different emphasis on teaching methods may be attributed to differences in cultures, policies, and school conditions [19].

### Assessment

#### Assessment methods

The assessment methods are related to the education forms (Table 2). For theoretical courses, educators employed the following: (1) test on concepts [16, 17] – a Brazilian dental school set an exam about basic concepts of dental biomaterial at the end of classes [16]; (2) article presentation [16] – students need to search articles on the assigned topic and present the articles as well as their perception of search methodologies, result translation, and critical reading skills in a seminar [16]; and (3) questionnaire [14–18]. The assessment methods of experimental skills training comprised the following: (1) exam [16]; (2) experimental report [16]; and (3) questionnaire [16]. Most research projects arranged the assessment at the end of the project, including: (1) submission of a thesis [13, 19, 22], which is the most popular and basic form of assessment. Details of the project report were well specified in several Swedish dental schools, such as page numbers, structure, layout, references, etc. [19]; (2) oral presentations or meetings [11, 13, 18] – the verbal presentation at the University of Manitoba was styled after an MSc thesis defence, where students summarized their findings and several professors provided oral feedback [13]. The Student Research Club (SRC) of Istanbul University held annual meetings where students gave 10- to 15-min speeches on their research. Additionally, a booklet containing all the project abstracts was distributed to participants, which promoted academic exchanges [11]; (3) competitions [18, 27] – in South Africa [18], undergraduates were awarded in Colgate Undergraduate Competition based on the project quality and their insights shown in the questioning part; (4) questionnaire [11, 15, 18, 21–23]; and (5) combination of the above methods [13, 18, 22, 23].

Nevertheless, considering the assessment time point, the majority of studies chose summative assessments [11, 13–15, 17, 19–23, 27, 29], while the others chose formative assessments [16, 18]. Among all the included studies, only one study mentioned midterm assessment [23]. Most projects mainly relied on mentors’ supervision during implementation, which may account for the abortion of some student research projects. Moreover, ambiguous assessment indicators of midterm supervision may not truly reflect the training outcome or the achievement of educational goals.

#### Assessment indicators

Targeting the educational goals, specific indicators were adopted according to assessment methods and the feasibility of indicator collection (Table 2): (1) The problem discovery ability can be assessed by self-assessment [11, 14, 22, 23] or evaluated along with other research abilities. For example, the number of funded projects can reflect both the ability of problem discovery and project design [11, 23]. (2) The literature retrieval ability can be evaluated through self-assessment [11, 17, 21]. (3) The research design capability can be depicted by the number of applied research funds [11, 23] and self-assessment [11]. (4) The ability of experimental operation can be assessed through experiment course scores [16, 23], experimental reports [16], and self-assessment [11, 14, 23]. (5) The scientific report writing ability can be evaluated by indicators, such as the number of published papers [23], research competition awards [27], and self-assessment [11, 14]. (6) Students’ interest in scientific research can be estimated through (a) instant feedback, such as the number of participants [13] and attendance rate [23]. (b) long-term influence, including future career choices and talent retention [11, 13, 18, 23, 29].

#### Summary

The assessment system varies across studies. Regretfully, imperfection of the current assessment is observed as they are incomprehensive and immethodical. The lack of assessment of overall education goals is manifested as the ignorance of one or several aspects of education goals. In addition, the neglect of mid-term assessment is common in the included studies. This leads to difficulties in evaluating the overall performance and comparing the effectiveness between studies and programs. Hence, a systemic and comprehensive assessment system based on education goals should be established to monitor the outcome of UDBRE for timely adjustment and long-term tracking.

### Outcomes

Existing reports uncover the education achievements of UDBRE, including target research ability development and scientific interest promotion (Table 3).

**Table 3:** Summary of dental basic research education outcomes of included literatures

Publication	Dental Basic Research Education outcome						
	Basic research question discovery ability	Literature retrieval ability	Research design capability	Experimental techniques	Scientific report writing ability	Promoting scientific interest	Other aspects
Scott, 2008 [27]	/	/	/	/	/	1. The number of participants increased from 1 (1980) to 11 (2005). 2. 14% of graduates remained as faculty members, 31.5% of graduates continued pursuing higher education positions.	1. The mean GPA was 3.42 and 3.14 for participants and non-participants. 2. Five valedictorians were members of the BSc Dent program for their overall outstanding performance.
Grossman, 2009 [18]	/	/	/	/	/	1. 44% of interviewees were satisfied with the research experience. 2. 92% thought research was important. 3. 34% agreed they would do research in the future.	In three out of four surveyed schools, over half of the students were unlikely to do research in the future.
Guven, 2011 [11]	The funded project number increased from 16 (1993) to 25 (2008).	1. Club members strongly agreed that they learned literature retrieval. 2. They agreed they had a reflection on scientific progress.	1. The funded project increased from 16 (1993) to 25 (2008) 2. Participants had independent inquiry skills.	Club members agreed that they had laboratory experience.	Club members strongly agreed that they developed the ability of data presentation and analysis.	1. Club members strongly agreed they were willing to do postgraduate research. 2. 74 SRC members continued studying at Istanbul University in the last 5 years. 3. 31% of teaching assistants were former club members.	1. Students had higher GPA after joining the club (3.22 ± 0.33 V.S. 2.90 ± 0.36). 2. Members had a higher GPA than non-members (3.05 ± 0.44 V.S. 2.55 ± 0.42).
Jeelani, 2014 [14]	59.2% of medical and dental students knew how to design and complete a study.	/	/	/	1. 46.7% of students knew how to write articles. 2. 17.7% of students knew the procedure of publication.	68.7% of surveyed medical and dental students had participated in the research.	/
Ping, 2015 [15]	/	/	/	/	/	1. 54% of surveyed dental students had participated in the research. 2. 73% of surveyed students were interested in research, but senior one had less research interest.	Half of the non-participants admitted that they had no interest in scientific research.
Habib, 2018 [29]	/	/	/	/	/	The survey showed that students had mediocre attitude toward scientific research.	/
Nieminen, 2020 [17]	/	80% of students had good or passable literature retrieval skills.	/	/	/	/	/
Otuyemi, 2020 [22]	45.2% of interviewees chose the research project topic individually whilst 20.4% were changed by supervisors.	/	/	/	/	1. Almost half of the students were satisfied with the final topic. 2. 26.6% of students gained confidence in research after the research project.	/
Yu, 2020 [23]	1. The basic research projects per year increased from 2 (2007) to 7 (2017). 2. The multidisciplinary project’s rate increased from 0 (2007) to 33.3% (2017).	/	The number of students per funded project decreased from 6.25 (2007) to 3.33 (2017).	Students strongly agreed they obtained experimental skills (4.00 ± 0.80).	The participants published more articles (1.62 ± 1.41 V.S. 1.31 ± 0.75 ) during the post-graduate period (*P* = 0.025).	1. The attendance rate of the research program increased from 36.84 to 90% 2. Students showed a certain satisfaction level (VAS^a^ score = 72.36 ± 20.37).	1. Participants had a higher GPA than non-participants (3.41 ± 0.02 V.S. 3.21 ± 0.04, *P* < 0.001) . 2. Participants won more honor rolls per student (0.53 ± 0.07 V.S. 0.30 ± 0.06, *P* < 0.05).
Scott and de Vries, 2008 [13]	/	/	/	/	Some students won prizes in research competitions.	/	/
Kyaw, 2018 [21]	/	51.2% of students believed they can appraise literature to a certain degree.	/	/	/	83.3% of interviewed medical and dental students had moderate attitudes toward scientific research.	/
Costa-Silva, 2018 [16]	/		/	1. Experiment group had higher course scores. 2. Most groups chose the correct test cell and protocol.	Participants had more content of methodology, concepts, and literature support on the experimental report.	/	/
Franzen, 2014 [20]	/	/	Students used various research methods, like quantitative, laboratory, and review methods.	/	/	/	/

*Abbreviations*: *GPA* Grade point average, *VAS* Visual analogue scale

^a^VAS score shows the degree of satisfaction. It ranges from 0 to 100

#### Development of target research abilities

(1) The problem discovery ability: Nigerian educators discovered that 45.2% of undergraduates chose the research topic by themselves [22]. (2) The literature retrieval ability: SRC participants strongly agreed that the program developed their experience of searching archives [11]. Likewise, Nieminen reported that almost 80% of undergraduates perceived to have good or passable literature retrieval skills after compulsory information retrieval lessons [17]. (3) The research design capability: Yu’s study revealed that the number of funded research projects has increased in the past 11 years, from 1 ~ 2 projects per year (2007–2011) to 7 projects per year (2017) [23]. Similarly, Guven’s study showed growing trend of funds and the participants agreed that they developed better research planning and independent inquiry skills during the research [11]. (4) The ability of experimental operation: A Brazilian study showed that compared with students who only participated in theoretical courses, the average course scores of PBL participants were slightly higher (7.8 ± 1.2 and 7.2 ± 1.6, respectively). Significantly more content of methodology and scientific literature support was detected in the experimental report of PBL participants [16]. In Yu’s and Guven’s study, the students believed that UDBRE helped to obtain experimental techniques [11, 23]. (5) The scientific report writing ability: A study showed that UDBRE participants published significantly more articles (1.62 ± 1.41) than nonparticipants (1.31 ± 0.75) during the postgraduate period [23]. UDBRE participants from the University of Manitoba won first place in a scientific competition (Canadian Association for Dental Research) for their outstanding scientific work [27]. Analogously, SRC participants agreed that the program strengthened their analytical skills as well as their ability to present research results and therefore formed a better basis for postgraduate studies [11]. However, only 46.7% of surveyed Pakistani medical and dental undergraduates claimed to know how to write articles. Few students (17.7%) acquired knowledge of the procedure of publication of articles, indicating that these studies scarcely emphasized the cultivation of thesis writing [14].

#### Students’ interest in scientific research

UDBRE has both short-term and long-lasting effects on students’ interest in scientific research (Table 3). (1) Instant feedback: UDBRE participants increased from one (1980) to 11 (2005) [13] in Scott’s study. Yu’s study found that the attendance rate of UDBRE increased from 36.84 to 90% and that students showed high satisfaction (VAS score = 72.36 ± 20.37) [23]. A South African study found that 92% of students realized the importance of basic research and 34% were willing to participate in research activities again [18]. The satisfaction rate of different Swedish dental schools varied from 26 to 50% [19]. Three studies reported that students with research experience possessed a mediocre attitude towards research and 75% did not gain confidence in research [21, 22, 29]. (2) Long-term influence: SRC members showed great willingness to pursue a Ph.D. degree. Furthermore,74 SRC members continued studying at Istanbul University from 2005 to 2009 and 31% of present teaching assistants were former SRC members [11]. Similarly, at the University of Manitoba, 31.5% of UDBRE graduates continued pursuing higher academic degrees and 17% of them obtained postgraduate programs [13]. However, Grossman found that in three out of four surveyed schools, over half of the students were unwilling to do research in the future [18].

#### Summary

Existing evidence (Table 3) shows that the UDBRE has reached certain education outcomes. UDBRE participants yielded satisfying advancements in targeted scientific research abilities. Undergraduates expressed a high degree of satisfaction with UDBRE and interest in scientific research and demonstrated more willingness to continue their postgraduate studies and academic careers. Few students conveyed negative attitudes [15, 18], who may encounter difficulties (conflict with clinical learning, failure in the experiment, lack of guidance, etc.). This arouses educators’ concern about barriers to UDBRE and reminds educators to offer guidance and assistance to improve the UDBRE program timely.

### Challenges

For current UDBRE deficiency, apart from subjective design reasons, objective obstacles cannot be neglected (Table 2), including excessive curriculum burden [10, 14, 29], shortage of academic faculty, and mentorship [6, 15, 18, 23, 29], insufficient financial support [10, 14, 18, 21, 23], and deficiency in research methodology and background knowledge [15, 17, 21, 23, 29]. Reflections and possible solutions are provided in some publications [10, 14, 15, 18, 23].

#### Excessive curriculum burden?

UDBRE may aggravate the heavy burden of dental clinical courses. In Pakistan, 91.9% of medical and dental undergraduates complained about the heavy curriculum load [14]. Twenty-two percent of students in South Africa [18] and 12% in China [23] admitted the conflict of study time and research time. Some studies observed a lack of interest and initiative towards scientific research due to the heavy load of time-and-energy-consuming dental clinical curricula [10, 29]. A survey demonstrated that only 34% of interviewees were sure to attend research even if it was voluntary [18].

In fact, follow-up studies on grade point average (GPA) [11, 13, 23] and scholarship [13, 23] showed that UDBRE had no negative impact, but rather a positive effect on the dental clinical study (Table 3). In Canada, UDBRE participants showed similar baseline GPAs as nonparticipants, while they gained significantly higher total GPAs upon graduation (3.42 ± 0.41 and 3.14 ± 0.44, respectively) [13]. Similar GPA comparison outcomes were observed by Guven (3.05 ± 0.44 and 2.55 ± 0.42, respectively, *P* < .001) [11] and Yu (3.41 ± 0.02 and 3.21 ± 0.04, respectively, *P* < .001) [23]. Moreover, 20% of outstanding graduates at Manitoba University have participated in UDBRE [13]. Likewise, Yu observed that UDBRE participants won significantly more Honor Rolls awards per student (0.53 ± 0.07) than nonparticipants (0.30 ± 0.06) [23].

These results indicate that students are capable of coping with such pressure, rather than it adversely affecting their study (Table 3). Therefore, educators should offer psychological guidance to release pressure, and optimize curriculum design to control time occupation. This provides an opportunity for universities to integrate the UDBRE into the undergraduate curriculum, sort out and optimize all existing undergraduate courses, integrate repeated lessons, and condense into a more reasonable undergraduate curriculum system. Flexibly setting primary and intermediate educational goals, adopting adjustable teaching methods by integrating core curriculum and extra curriculum, and the early exposure to UDBRE serve as alternatives to avoid time conflict.

#### Tutors shortage and insufficient guidance?

The lack of academic faculty is mostly mentioned in the UDBRE literature [6, 18, 29]. From 2004 to 2005, there were 250 unfilled faculty positions in dental schools in the USA [30]. The same applies to South Africa [18] and the United Kingdom [6].

Moreover, quite a few studies reflect the insufficient guidance of faculty [15, 18, 23]. Grossman [18] found that nearly one fifth of students felt inadequate supervisory assistance. These educational skills were lacking at the beginning of most junior assistant professors’ careers [23]. This could be a serious problem. Supervisors lacking mentorship had a negative impact on students’ research experience [31].

Several measures solve the shortage, including: (1) to increase the salary of research faculty; (2) to expand the faculty troop by recruiting young doctors, postdoctoral fellows, and even academic tutors from other disciplines; (3) to integrate student projects into teachers’ research fields, which promotes more detailed and professional guidance from tutors and releases tutors’ understaffed situations with undergraduates’ assistance; and (4) to provide mentor training courses to junior tutors.

#### Lack of financial support?

Both students and faculty acknowledged that funds and financial support were essential for student research projects [23]. However, 86.9% and 92.6% of medical and dental students, respectively, faced fund shortages in Pakistan [14]. The government, universities, and dental schools are indispensable in providing financial support and an academic environment to enable the sustainable operation of the UDBRE [23].

#### Inadequate basic research skills and background knowledge?

A shortage of basic research skills and background knowledge led to difficulty in the initiation and a decrease in initiative [15, 29]. Undergraduates, especially freshmen, spend more time absorbing background knowledge and methodology of scientific research [15]. Even fourth-year dental students exhibited insufficient research knowledge and unsatisfactory information retrieval ability [17].

Moreover, most students are only familiar with the background knowledge of a certain topic notwithstanding multidisciplinary research is a new trend in basic research. Yu recorded an increasing proportion of multidisciplinary projects from 0 (2007) to a maximum of 55.56% (2015). In addition, both dental faculty and students were aware that cross-departmental training was essential to completing the research project, with 8.22% of interviewees calling on facilitating multidisciplinary cooperation [23].

Thus, undergraduates should attend theoretical and experimental courses to hone basic research skills before undertaking a research project. Nevertheless, teaching comes with difficulties while the integration of basic research experimental training into the curriculum system may serve as a solution. For example, microbial-related experiment training can be integrated into dental microbiology courses. Supplementary education can also be used to provide further study opportunities.

In response to the lack of background knowledge and the trend of multidisciplinary research, the authors proposed a new UDBRE component--rotation in different research departments, where students can practice basic research skills and gain background knowledge of different research fields.

#### Summary

There are still various difficulties in the implementation of UDBRE, as well as corresponding solutions (Table 2). It is necessary to optimize the UDBRE system, so that students can smoothly start research projects rather than encountering difficulties and losing interest. Measures are also needed to enhance teachers’ responsibility and interest.

### Implications for undergraduate dental basic research education

Although UDBRE has not yet been popularized worldwide, the establishment of UDBRE has been explored by some countries, and UDBRE programs have recently increased. Through systematically reviewing these useful explorations and experiences, some enlightening implications were obtained.

**Implications for dental education goals**. There is an urgent need for compound talent with both clinical skills and basic research capacity. Even for clinical dentists, critical thinking and evidence-based medical thinking are also beneficial. The education of common research essence in UDBRE, such as research question discovery, literature retrieval, research design, and report writing, can favour dentists in their future clinical careers. Equipping students with primary but overall scientific research abilities so that students can develop critical thinking and form evidence-based minds is of great educational significance. Specific goals of UDBRE programs are proposed, but divergence exists within studies (Table 2). These specific goals can be summarized as “cultivating dental research talent with basic research capabilities and strong scientific interests”. Therefore, promoting scientific interest and cultivating five major research abilities, including basic research question discovery, literature retrieval, research design, experimental operation, and scientific report writing may serve as ideal objectives of UDBRE. Clarifying the education target may help dental schools design specific education methods to fulfil the goals. Setting primary and intermediate goals can help reduce students’ workload and increase the feasibility of UDBRE.

**Fig. 2:**
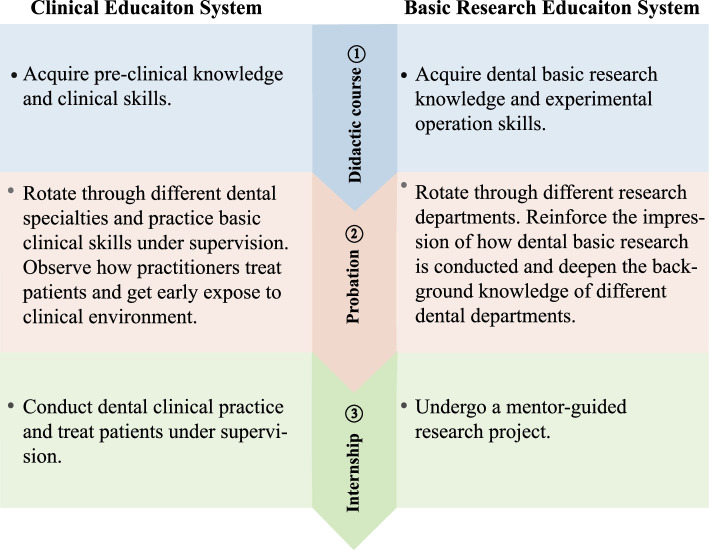
The three-stage UDBRE system mimicking the dental clinical training system. Since the education model of UDBRE is still under exploration and the dental clinical training system is relatively mature, we have attempted to arrange and classify the training contents of UDBRE into three stages mimicking the current clinical training system, including didactic course, probation, and internship

**Fig. 3:**
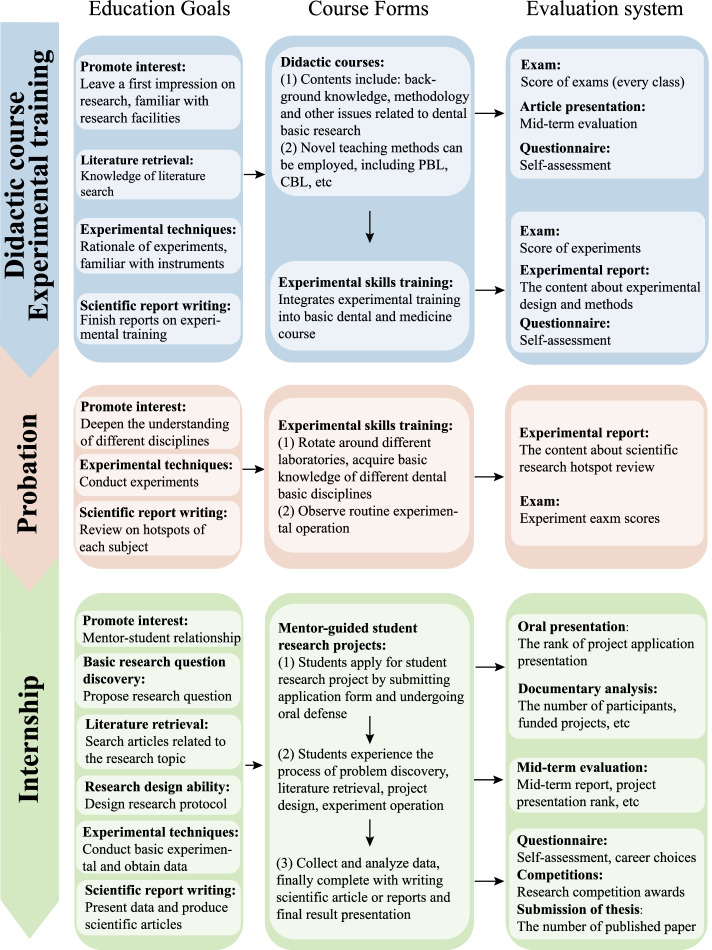
Detailed information of an example of UDBRE system which consisted of three stages. The prospective education goals, course forms, and assessment system of the undergraduate dental basic research education (UDBRE) program are listed in detail along the timeline

**Implications for the dental course system**. As a more skill-based course, there may be some concerns about dental basic research education in increasing the course burden and clinical study outcome. From the review outcomes and experience in carrying this course, it seems that UDBRE had no negative impact, but rather a positive effect on dental clinical study; students tend to have higher clinical-related course GPAs. This further confirms the necessity and feasibility of vertically integrating this system. Dental basic research education is systematic work, and the UDBRE system can be early, continuous, and long-term and be carried out simultaneously with clinical education in a vertically integrated way [32]. Mimicking the mature dental clinical training system, this study attempts to arrange and classify the training contents of UDBRE into three stages (Figs. 2 and 3): (1) Didactic course and experimental training period. In the first stage, students are expected to acquire the necessary basic research theory and skills, which may overcome the barrier of inadequate basic research skills and help students start the research practice (Fig. 3). Tables 4 and 5 lists examples of optimized and integrated curriculum settings. (2) Probationary period. Students with cumulative dental research knowledge and skills are introduced to different research departments as clinical rotations. The early direct exposure to different dental basic research departments enables reinforcement of impressions on how dental basic research is conducted and deepening of the knowledge of different disciplines. (3) Internship period. During this period, students shift from being passive audiences to active participants by undergoing a mentor-guided research project in a similar manner as the clinical internship. At the end of this preparatory stage, students are encouraged to finish an undergraduate research thesis. It should be noted that this teaching system is only one example (which has been carried out in our school), and different dental schools can adjust to the proper UDBRE system for them.

**Table 4:** An example of didactic courses at the first stage of the UDBRE system. According to the cultivation of the five research abilities in the education goals, the contents generally include five sessions: (1) formulating a good research question; (2) answering the research question; (3) seeking funds; (4) presenting findings; (5) other issues during the scientific inquiry

Education Module	Didactic course content
**Module 1. Formulate a good research question**	Introduction to major topics in dental basic research
	Literature review skill
	Principles and methods for formulating a good research question
**Module 2. Answer the research question**	Literature search strategies: searching and evaluating literature
	Introduction to research designs & basic research skills and techniques
	Accessible sources for research
	Data management and analysis
	Safety issues in laboratories
**Module 3. Present research findings**	Introduction to paper types
	Process of writing and getting published
	Participation in academic conferences
	Studying abroad & visiting scholarship
	Patent application
**Module 4. Seek research funding**	Introduction to fund category
	Grant application
**Module 5. Other issues during scientific inquiry**	Dealing with negative emotions and stress
	Academic misconduct and integrity education

**Table 5:** An example of experimental training at the first stage of the UDBRE system

Specialty	Requisite research skills
**Cell biology**	Isolation and purification of cells and their components; cell culture; use of optical microscope and photomicrography; in situ hybridization; preparation of culture medium;
**Molecular biology**	Use of Micropipette; western blot; polymerase chain reaction (PCR); agarose gel electrophoresis; extraction of genomic DNA from eukaryotic cells; restriction enzyme digestion;
**Oral microbiology**	Isolation and culture of bacteria; medium transfer technique; common bacteria identification and staining methods; drug sensitivity test; germicidal test; use of oil immersion objective; preparation and inoculation of culture medium;
**Dental materials**	Methods for materials component analysis (infrared spectrometry, chromatography, mass spectrometry) and materials surface analysis (use of optical microscopic, scanning electron microscope, atomic force microscope); tests for bonding, curing, and mechanical property, deformation, hardness, and fluidity.
**Oral and maxillofacial oncology**	Fluorescent quantitative PCR; flow cytometry; immunofluorescence technique; immunohistochemical technique; identification of protein by mass spectrometry;
**Oral and maxillofacial histology and pathology**	Making paraffin sections; cell staining (HE staining; immunofluorescence staining);

**Implications for the assessment system**. Concerning the common neglect of mid-term assessment and focus on certain research abilities, we propose that assessments should be targeted at overall educational goals and should be conducted not only at the end of each stage but throughout the whole period so as to adjust and formulate individualized training plans according to feedback. We have attempted to arrange and classify the assessment system of UDBRE, which can be found in Fig. 3.

#### Future prospects

These implications enlighten a scientific, gradual, and long-term UDBRE system (Fig. 3). Undergraduates can be exposed early to dental basic science to maximize research experience and the opportunity to conduct publishable research. In support of further advancement, it is encouraged that universities report comprehensively in a structured way on their UDBRE programs to allow comparison and reproduction. With the development of the UDBRE system, it is of interest in the future to set up a new degree program that focuses on dental basic research for students with dental clinical medical backgrounds.

## Data Availability

Data sharing is not applicable to this article as no datasets were generated or analysed during the current study.

## Abbreviations

UDBRE: Undergraduate Dental Basic Research Education
ERIC: Education Resources Information Center
GPA: Grade point average
VAS: Visual analogue scale

## References

[1] Bush V. Science: The Endless Frontier A Report to the President by Vannevar Bush, Director of the Office of Scientific Research and Development. United States Government Printing Office. https://www.nsf.gov/about/history/nsf50/vbush1945_content.jsp. Published 1945. Accessed 13 Nov 2009.

[2] Slavkin HC (2017). The impact of research on the future of dental education: how research and innovation shape dental education and the dental profession. J Dent Educ.

[3] Selwitz RH, Ismail AI, Pitts NB (2007). Dental caries. Lancet.

[4] Sandy JR, Farndale RW, Meikle MC (1993). Recent advances in understanding mechanically induced bone remodeling and their relevance to orthodontic theory and practice. Am J Orthod Dentofac Orthop.

[5] Iacopino AM, Lynch DP, Taft T (2004). Preserving the pipeline: a model dental curriculum for research non-intensive institutions. J Dent Educ.

[6] Rushton VE, Horner K (2008). Academic dentistry. J Dent.

[7] Istrate EC, Slapar FJ, Mallarapu M, Stewart DCL, West KP (2021). Dentists of tomorrow 2020: an analysis of the results of the 2020 ADEA survey of U.S. dental school seniors. J Dent Educ.

[8] D'Souza RN, Colombo JS, Embree MC, Myers JM, DeRouen TA (2017). Our essential and endangered dentist-scientist workforce. JDR Clin Trans Res.

[9] Gies WJ (1926). Dental education in the United States and Canada. A report to the Carnegie Foundation for the advancement of teaching. J Am Coll Dent 2012.

[10] Divaris K, Barlow PJ, Chendea SA, Cheong WS, Dounis A, Dragan IF, Hamlin J, Hosseinzadeh L, Kuin D, Mitrirattanakul S (2008). The academic environment: the students' perspective. Eur J Dent Educ.

[11] Guven Y, Uysal O (2011). The importance of student research projects in dental education. Eur J Dent Educ.

[12] Nalliah RP, Lee MK, Da Silva JD, Allareddy V (2014). Impact of a research requirement in a dental school curriculum. J Dent Educ.

[13] Scott JE, de Vries J, Iacopino AM (2008). 25-year analysis of a dental undergraduate research training program (BSc dent) at the University of Manitoba Faculty of dentistry. J Dent Res.

[14] Jeelani W, Aslam SM, Elahi A (2014). Current trends in undergraduate medical and dental research: a picture from Pakistan. J Ayub Med Coll Abbottabad.

[15] Ping W (2015). Dental undergraduate students' participation in research in China: current state and directions. Eur J Dent Educ.

[16] Costa-Silva D, Cortes JA, Bachinski RF, Spiegel CN, Alves GG (2018). Teaching cell biology to dental students with a project-based learning approach. J Dent Educ.

[17] Nieminen P, Uma E, Pal S, Laitala ML, Lappalainen OP, Varghese E (2020). Information retrieval and awareness about evidence-based dentistry among dental undergraduate students-a comparative study between students from Malaysia and Finland. Dent J.

[18] Grossman ES, Naidoo S (2009). Final-year south African dental student attitudes toward a research component in the curriculum. J Dent Educ.

[19] Franzén C, Brown G (2013). Undergraduate degree projects in the Swedish dental schools: a documentary analysis. Eur J Dent Educ.

[20] Franzen C (2014). The undergraduate degree project - preparing dental students for professional work and postgraduate studies?. Eur J Dent Educ.

[21] Kyaw Soe HH, Than NN, Lwin H, Nu Htay MNN, Phyu KL, Abas AL (2018). Knowledge, attitudes, and barriers toward research: the perspectives of undergraduate medical and dental students. J Educ Health Promot.

[22] Otuyemi OD, Olaniyi EA (2020). A 5-year retrospective evaluation of undergraduate dental research projects in a Nigerian University: Graduates' perceptions of their learning experiences. Eur J Den Educ.

[23] Yu W, Sun Y, Miao M, Li L, Zhang Y, Zhang L, et al. Eleven-year experience implementing a dental undergraduate research program in a prestigious dental school in China: lessons learned and future prospects. Eur J Den Educ. 2020. 10.1111/eje.12598. 10.1111/eje.12598 32967058

[24] Administrative measures for the National Innovation and Entrepreneurship Training Program for College Students. Ministry of Education of the People’s Republic of China. https://www.moe.gov.cn/jyb_xwfb/gzdt_gzdt/s5987/201907/t20190731_393103.html. Published 2019. Assessed 4 Apr 2021

[25] Wu ZY, Zhang ZY, Jiang XQ, Guo L (2010). Comparison of dental education and professional development between mainland China and North America. Eur J Den Educ.

[26] Huang C, Bian Z, Tai B, Fan M, Kwan CY (2007). Dental education in Wuhan, China: challenges and changes. J Dent Educ.

[27] Scott JE (2008). Undergraduate experience in dental research: the bachelor of science (dentistry) program at the University of Manitoba. J Can Dent Assoc.

[28] Tricco AC, Lillie E, Zarin W, O'Brien KK, Colquhoun H, Levac D, Moher D, Peters MDJ, Horsley T, Weeks L (2018). PRISMA extension for scoping reviews (PRISMA-ScR): checklist and explanation. Ann Intern Med.

[29] Habib SR, AlOtaibi SS, Abdullatif FA, AlAhmad IM (2018). Knowledge and attitude of undergraduate dental students towards research. J Ayub Med Coll Abbottabad.

[30] Chmar JE, Weaver RG, Valachovic RW (2006). Dental school vacant budgeted faculty positions: academic year 2004-05. J Dent Educ.

[31] Chang Y, Ramnanan CJ (2015). A review of literature on medical students and scholarly research: experiences, attitudes, and outcomes. Acad Med.

[32] Brauer DG, Ferguson KJ (2015). The integrated curriculum in medical educatioSn: AMEE guide no. 96. Med Teach.

